# Intracellular calcium release modulates polycystin-2 trafficking

**DOI:** 10.1186/1471-2369-14-34

**Published:** 2013-02-11

**Authors:** Ayako Miyakawa, Cristián Ibarra, Seth Malmersjö, Anita Aperia, Peter Wiklund, Per Uhlén

**Affiliations:** 1 Department of Medical Biochemistry and Biophysics, Karolinska Institutet, SE-171 77, Stockholm, Sweden; 2 Department of Molecular Medicine and Surgery, Karolinska University Hospital, SE-171 76, Stockholm, Sweden; 3 Department of Woman and Child Health, Astrid Lindgren Children’s Hospital Q2:09, Karolinska University Hospital, SE-171 76, Stockholm, Sweden

**Keywords:** Polycystin-2, Protein trafficking, Calcium signaling, Kidney cells, Autosomal dominant polycystic kidney disease

## Abstract

**Background:**

Polycystin-2 (PC2), encoded by the gene that is mutated in autosomal dominant polycystic kidney disease (ADPKD), functions as a calcium (Ca^2+^) permeable ion channel. Considerable controversy remains regarding the subcellular localization and signaling function of PC2 in kidney cells.

**Methods:**

We investigated the subcellular PC2 localization by immunocytochemistry and confocal microscopy in primary cultures of human and rat proximal tubule cells after stimulating cytosolic Ca^2+^ signaling. Plasma membrane (PM) Ca^2+^ permeability was evaluated by Fura-2 manganese quenching using time-lapse fluorescence microscopy.

**Results:**

We demonstrated that PC2 exhibits a dynamic subcellular localization pattern. In unstimulated human or rat proximal tubule cells, PC2 exhibited a cytosolic/reticular distribution. Treatments with agents that in various ways affect the Ca^2+^ signaling machinery, those being ATP, bradykinin, ionomycin, CPA or thapsigargin, resulted in increased PC2 immunostaining in the PM. Exposing cells to the steroid hormone ouabain, known to trigger Ca^2+^ oscillations in kidney cells, caused increased PC2 in the PM and increased PM Ca^2+^ permeability. Intracellular Ca^2+^ buffering with BAPTA, inositol 1,4,5-trisphosphate receptor (InsP_3_R) inhibition with 2-aminoethoxydiphenyl borate (2-APB) or Ca^2+^/Calmodulin-dependent kinase inhibition with KN-93 completely abolished ouabain-stimulated PC2 translocation to the PM.

**Conclusions:**

These novel findings demonstrate intracellular Ca^2+^-dependent PC2 trafficking in human and rat kidney cells, which may provide new insight into cyst formations in ADPKD.

## Background

Autosomal dominant polycystic kidney disease (ADPKD) is the most commonly inherited monogenetic disease, affecting more than 1 in 1000 live births, causing renal failure [1]. ADPKD is caused by mutation in two associated proteins, polycystin-1 or −2 (PC1 and PC2), which are essential for the formation and maintenance of a proper structure of the renal tubule. These mutations in PC1 and PC2 are responsible for approximately 85% and 15% of all ADPKD cases, respectively [2]. It is well established that PC2 acts as an ion channel permeable to calcium ions (Ca^2+^) [3]. Interestingly, loss of PC2 channel function and subsequent impaired Ca^2+^ signaling may contribute to ADPKD pathogenesis [4]. A controversial aspect of PC2-mediated signaling in ADPKD is whether this protein is expressed in the endoplasmic reticulum (ER) or in the plasma membrane (PM). The amount of PC2 present in the ER, where it functions as a Ca^2+^-activated intracellular Ca^2+^ release channel [3], is significantly larger than the amount of PC2 expressed in the PM, where it functions as a non-selective cation channel [5,6]. In addition, PC2 has also been documented in the primary cilium of kidney epithelial cells, where it contributes to the mechano-sensing machinery by mediating Ca^2+^ entry in response to flow rate changes [7]. Nevertheless, the exact function and mechanism of PC2 activation in the cilium and/or other subcellular organelles remain largely unknown.

Ca^2+^ signaling is a vital mechanism in many cell types, controlling diverse cellular processes, such as: secretion, mechano-transduction, cell death, gene expression, or proliferation (for review see [8]). Under certain conditions, via a sophisticated interplay between Ca^2+^ channels and transporters located in the PM and/or on the membrane of internal organelles, such as the ER, sustained oscillatory Ca^2+^ signaling can occur [9]. These Ca^2+^ oscillations encode important information in their frequency and amplitude, which is decoded by cells using different Ca^2+^-effectors, such as protein kinases, phosphatases, proteases or transcription factors, which in turn regulate adaptive cellular responses. Intracellular Ca^2+^ signaling mishandling is involved in the pathogenesis or progression of several disease conditions and particularly in kidney disease, where intracellular Ca^2+^ signaling appears to be linked to cystic formation during ADPKD [10].

In the current study, we investigated the dynamic nature of PC2 localization in primary human and rat kidney proximal tubule cells. We found that PC2 translocates from a basal, reticular-like localization to a PM localization when cells were challenged with agents that raise the basal concentration of intracellular Ca^2+^. PC2 trafficking was inhibited either by intracellular Ca^2+^ release blockers or by inhibitors of key Ca^2+^-dependent proteins. Taken together, these results demonstrate that PC trafficking in kidney cells is regulated by intracellular Ca^2+^ and may contribute to the general understanding of ADPKD.

## Methods

### Cells cultures

Primary cultures of rat proximal tubule (rPT) cells were prepared as described previously [11]. Briefly, kidneys from 20-day-old female Sprague Dawley rats were used to prepare rPT cells. Cells were cultured in supplemented DMEM (20 mM Hepes, 24 mM NaHCO_3_, 10 mg/ml penicillin, 10 mg/ml streptomycin and 10% fetal bovine serum (FBS)) on glass coverslips for 48–72 h in 5% CO_2_ at 37°C. Cells were starved in 1% FBS and cultured in the absence of antibiotics for 24 h before the experiment. Primary cultures of human proximal tubule (hPT) cells were prepared as described. Briefly, approximately 10 g of fresh renal tissue, obtained from human nephrectomy samples, was dissected, and the obtained cortex was minced and then enzymatically digested with 1 mg/ml of type 4 collagenase. The resulting suspension was filtered and then centrifuged at 200 x g for 5 min. The pellet was washed three times with ice-cold Hank’s balanced salt solution and finally resuspended in DMEM/Ham’s Nutrient Mixture F12, supplemented with 10% FBS, insulin-transferrin-selenium, hydrocortisone and antibiotics. The final cell suspension was then plated onto coverslips coated with type IV collagen. All experiments were ethically approved by the Swedish Ethical Committee North, numbers 183/03 (rat) and 03–143 (human).

### Reagents

Reagents and concentrations were as follows: ouabain (1 μM for hPT cells and 100 μM for rPT cells since rodents are more resistant to ouabain than humans [12], Sigma-Aldrich), ATP (25 μM, Sigma-Aldrich), bradykinin (20 nM, Sigma-Aldrich), ionomycin (1 μM, Sigma-Aldrich), thapsigargin (1 μM, Sigma-Aldrich), bis(2-aminophenoxy)ethane tetraacetic acid (BAPTA, 10 μM, Molecular Probes), 2-aminoethoxydiphenyl borate (2-APB, 100 μM, Sigma-Aldrich), KN-93 (10 μM, Sigma-Aldrich), LY294002 (10 μM, Sigma-Aldrich), wortmanin (5 μM, Sigma-Aldrich), FK506 (20 nM, Sigma-Aldrich), Go6983 (1 μM, Sigma-Aldrich), calphostin (5 μM, Sigma-Aldrich), and cycloheximide (CHX, 100 μM, Sigma-Aldrich). Cells were treated with inducers of Ca^2+^ signaling (ouabain, ATP, bradykinin) 3 h prior immunostaining and with inhibitors 15 min prior Ca^2+^ signaling-inducers.

### Immunocytochemistry

Immunocytochemistry of PC2 in hPT and rPT cells was performed according to standard protocol. Cells were fixed with 4% paraformaldehyde for 15 min and then permeabilized for 10 min with 0.3% Triton X-100. After blocking with 1% BSA for 1 h, cells were incubated overnight at 4°C with anti-PC2 polyclonal antibodies (generously provided by Dr. Stefan Somlo, Department of Internal Medicine, Yale University School of Medicine, New Haven, CT, USA and Dr. Jing Zhou, Renal Division, Department of Medicine, Brigham and Women’s Hospital and Harvard Medical School, Boston, MA, USA) against amino acids 103 to 203 (YCB9) [13] or amino acids 44 to 62 [7] on the N-terminus, respectively. Both antibodies showed similar PC2 pattern in proximal tubule cells (Additional file 1: Figure S1). All images presented here are using the antibody against YCB9. Cells were incubated for 1 h at room temperature with Alexa488 fluorescent secondary antibody (1:500, Molecular Probes). Staining with only secondary antibody was used as control (Additional file 2: Figure S2). Slides were scanned with similar exposure time using a Leica TCS SP inverted confocal laser scanning microscope equipped with a 40x/1.4 NA oil-immersion objective.

### Calcium imaging

For Ca^2+^ experiments cells were loaded with 5 μM Fura-2/AM (Invitrogen) at 37°C for 1 h. Calcium measurements were performed at 37°C in a heated chamber (QE-1, Warner Instruments) with a cooled CCD camera (ORCA-ERG, Hamamatsu) mounted on an upright microscope (Axioskop 2 FS, Zeiss) equipped with a 40x/0.8 NA water dipping lens. Excitation at 340 and 380 nm was carried out with a monochromator (Polychrome IV, TILL Photonics). Devices were controlled and data were recorded and analyzed with the computer software MetaFluor (Molecular Devices). All experiments were performed in physiological buffer (100 mM NaCl, 4 mM KCl, 25 mM NaHCO_3_, 1.5 mM CaCl_2_, 1.1 mM MgCl_2_, 1 mM NaH_2_PO_4_, 10 mM D-glucose, and 20 mM HEPES, pH 7.4). An oscillating cell was defined as a cell that displayed two or more Ca^2+^ peaks, of which each peak’s amplitude was at least 10% over baseline. PM permeability was measured using the Fura-2 fluorescence quenching technique with Mn^2+^. Fura-2 was then excited at the Ca^2+^-dependent wavelength, 340 nm, and at the Ca^2+^-independent wavelength, 360 nm. The PM Ca^2+^ permeability was measured as the fluorescence decrease after adding 0.1 mM MnC1_2_ to the recording medium.

### Statistics

PM PC2-positive cells were counted and expressed as the percentage over the total number of cells. The percentage of PM-positive cells corresponding to the control condition was assigned a value of 1, and the different treatments were normalized over this value in order to express them as fold-values over control. Data are presented as mean ± SEM or as a representative result of at least three independent experiments. One-way ANOVA with a Bonferroni post hoc test was used and significance was accepted at *P* < 0.05.

## Results

### Subcellular localization of PC2 is dynamically regulated

**Figure 1:**
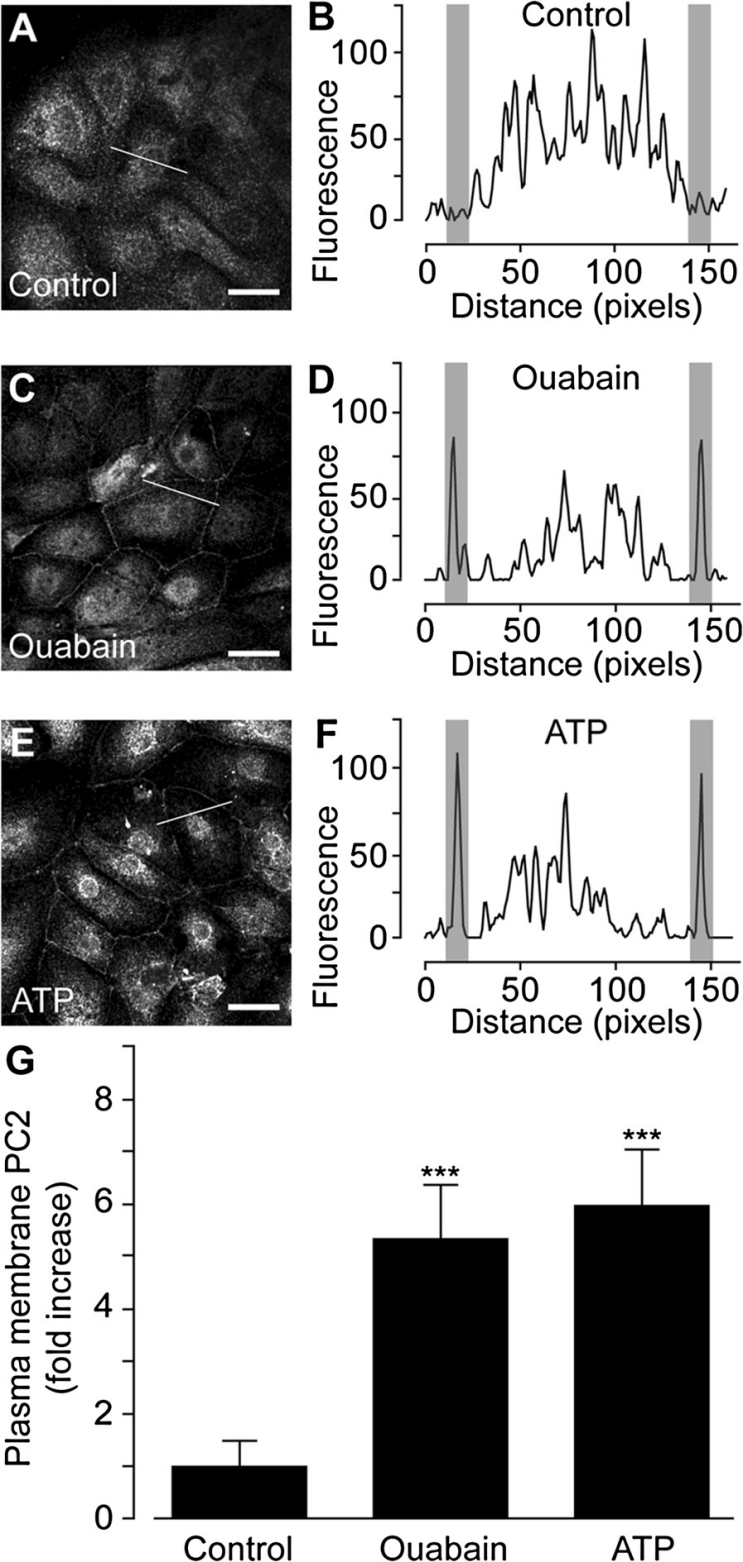
**PC2 trafficking to the PM in human proximal tubule (hPT) cells. (A**-**F).** Immunocytochemistry of PC2 in hRT cells treated with control **(A)**, 1 μM ouabain **(C)**, or 25 μM ATP **(E)**. Scale bars, 20 μm. Localization profile of PC2 along a line (as indicated) through the center of a single hPT cell treated with control **(B)**, ouabain **(D)**, or ATP **(F)**. Gray areas represent PM regions. **(G)** Quantitative analysis of a number of PM PC2-positive hRT cells following indicated treatments. Values are the mean ± SEM, and ***P < 0.001 vs. control.

The subcellular localization of the PC2 protein was studied using immunocytochemistry in human primary proximal tubule (hPT) cells prepared from nephrectomy patients, as described in Materials and methods. In un-stimulated hPT cells PC2 exhibited a cytoplasmic/reticular staining (Figure 1A), in good agreement with a previous report [3]. Plotting the localization profile along a line through an individual cell confirmed the cytoplasmic/reticular localization pattern (Figure 1B). To test the dynamic nature of PC2 localization, hPT cells were challenged with the steroid ouabain, known to induce intracellular Ca^2+^ oscillations in rat kidney cells [14,15]. When hPT cells were exposed to ouabain, a strong PC2 localization in the PM was observed (Figure 1C and D). To test whether this effect was due to an increase of cytosolic Ca^2+^, hPT cells were treated with nucleotide adenosine triphosphate (ATP), a well-established inducer of cytosolic Ca^2+^ signaling in renal cells [16]. hPT cells exposed to ATP exhibited a clear translocation of PC2 towards the PM (Figure 1E and F). Quantification analysis of *n* single-cells from *N* independent preparations showed that the number of hPT cells positive for PC2 in the PM was significantly increased 5.3 ± 1.0-fold by ouabain and 6.0 ± 1.1-fold by ATP (control *n* = 76 *N* = 6, ouabain *n* = 159 *N* = 7, ATP *n* = 70 *N* = 4) (Figure 1G).

**Figure 2:**
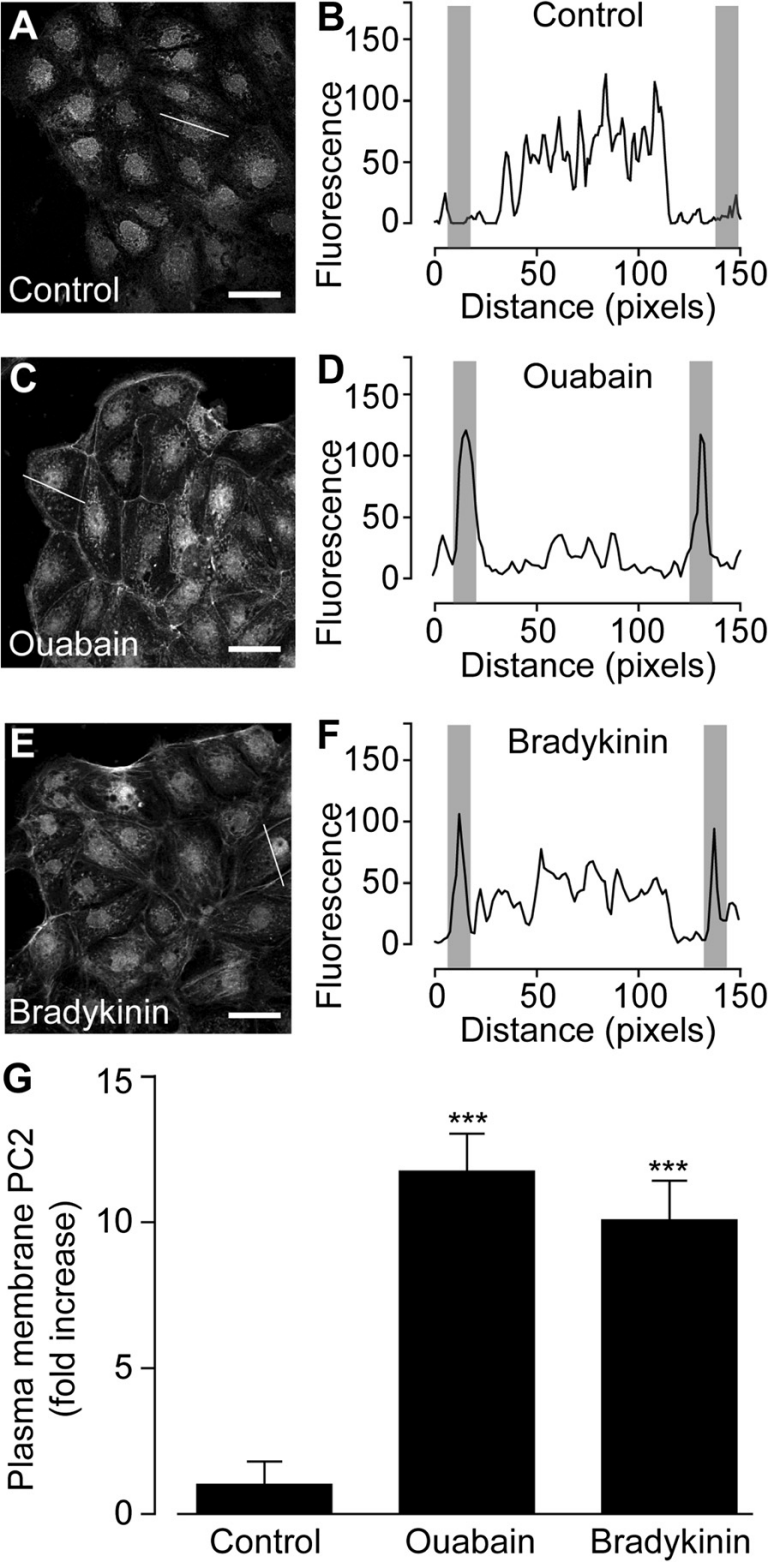
**PC2 trafficking to the PM in rat proximal tubule (rPT) cells. (A**-**F)** Immunocytochemistry of PC2 in rPT cells treated with control **(A)**, 100 μM ouabain **(C)**, or 20 nM bradykinin **(E)**. Scale bars, 20 μm. Localization profile of PC along a line (as indicated) through the center of a single rPT cell treated with control **(B)**, ouabain **(D)**, or bradykinin **(F)**. Gray areas represent PM regions. **(G)** Quantitative analysis of a number of PM PC2 positive rPT cells following indicated treatments. Values are the mean ± SEM and ***P < 0.001 vs. control.

Next, the dynamic localization pattern of PC2 in rat kidney cells was investigated. Immunocytochemistry experiments in primary cultures of rat proximal tubule (rPT) cells showed a cytoplasmic/reticular localization pattern of PC2 in un-stimulated cells (Figure 2A and B), in accordance with results from hPT cells. This basal PC2 localization was clearly translocated towards the PM when rPT cells were treated with ouabain (Figure 2C and D) or bradykinin (Figure 2E and F). Both ouabain and bradykinin are reported to induce a Ca^2+^ response in rPT cells [15]. Quantitative analysis showed that the number of rPT cells positive for PC2 in the PM was significantly increased 11.7 ± 2.1-fold by ouabain and 10.1 ± 2.2-fold by bradykinin (control *n* = 175 *N* = 8, ouabain *n* = 123 *N* = 7, bradykinin *n* = 104 *N* = 5) (Figure 2G).

**Figure 3:**
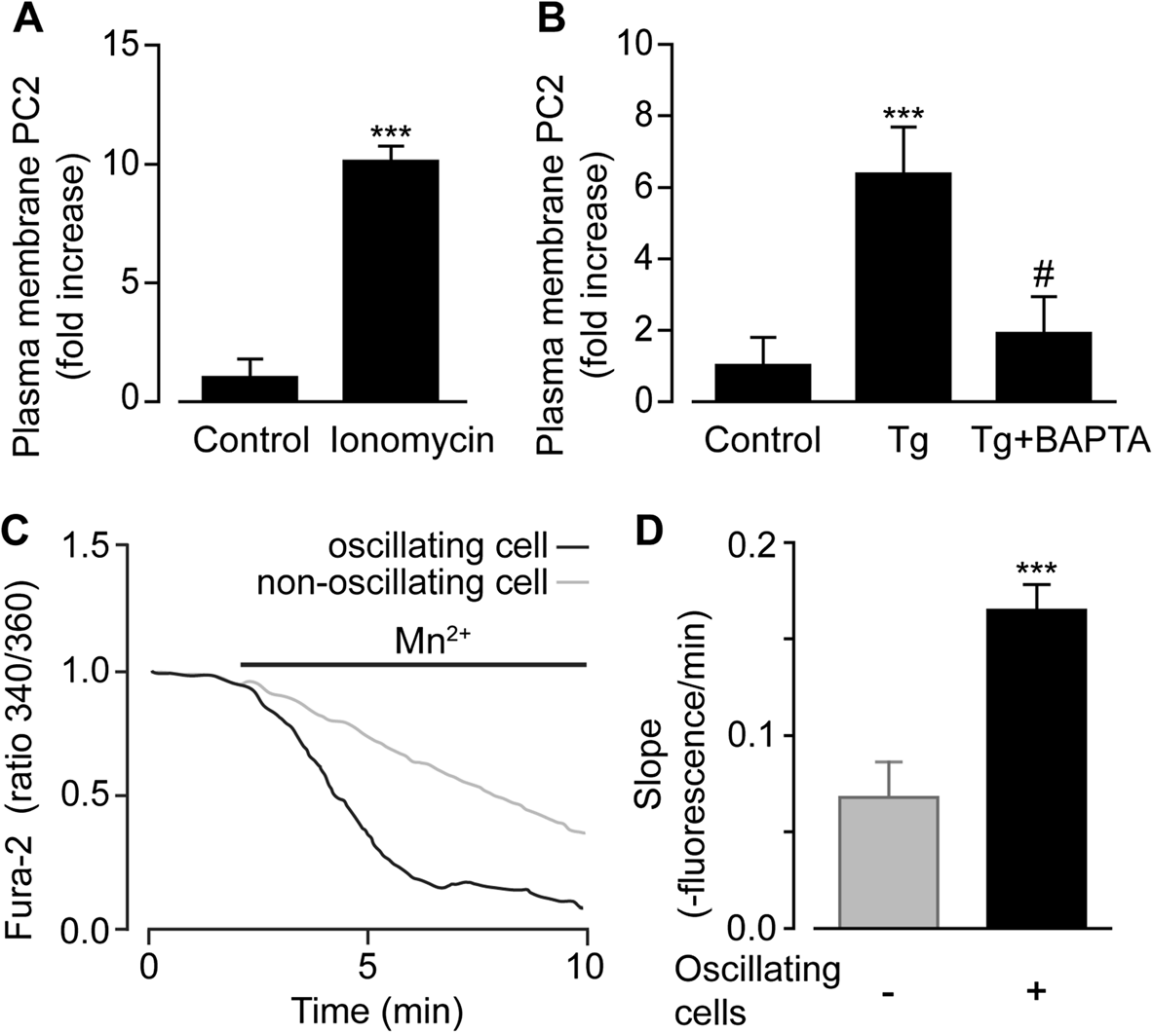
**Intracellular Ca**^**2+ **^**release mediates PC2 translocation to the PM. (A**-**B)** Quantitative analysis of a number of PM PC2-positive rPT cells following treatment with 1 μM ionomycin **(A)**, 1 μM thapsigargin (Tg) **(B)**, or thapsigargin with 10 μM BAPTA **(B)**. **(C)** Single cell Ca^2+^ recording of two rPT cells loaded with Fura-2/AM treated with 100 μM ouabain followed by sequential addition of 2 mM Mn^2+^ to the recoding medium. **(D)** Statistical analysis of slope decrease in non-oscillating (gray bar) and oscillating cells (black bar) in response to ouabain after Mn^2+^ addition to the recording medium. Values are the mean ± SEM, *P < 0.05, ***P < 0.001 vs. control, and #*P* < 0.001 vs. treatment.

To test whether an increase of the cytosolic Ca^2+^ concentration was sufficient to induce PC2-trafficking towards the PM, rPT cells were exposed to various well-known agents affecting the cellular Ca^2+^ machinery. First, a Ca^2+^ influx was induced by using the Ca^2+^ ionophore ionomycin. Ionomycin caused a significant 10.1 ± 0.6-fold increase of PC2 PM positive rPT cells (control *n* = 175 *N* = 8, ionomycin *n* = 139 *N* = 8) (Figure 3A). In another approach, a transient Ca^2+^ release from the ER was induced by using thapsigargin, an inhibitor of the sarcoplasmic/endoplasmic reticulum Ca^2+^ ATPase, which caused a raise in cytosolic Ca^2+^. Thapsigargin stimulated a significant 6.4 ± 1.3-fold increase of PC2 translocation to the PM in rPT cells (thapsigargin *n* = 73 *N* = 6) (Figure 3B). The stimulatory effect of thapsigargin on PC2 trafficking was 1.9 ± 1.0-fold and reversed in cells pre-incubated with BAPTA (thapsigargin + BAPTA *n* = 76 *N* = 7), an intracellular Ca^2+^ chelator (Figure 3B).

Taken together, these results demonstrate that cytosolic Ca^2+^ regulates PC2 trafficking in both human and rat kidney cells.

### Ca^2+^ channels in the PM are more abundant in Ca^2+^ oscillating cells

A functional study of the PM Ca^2+^ permeability was then conducted using the manganese (Mn^2+^) quenching technique, based on the higher affinity Fura-2 has for Mn^2+^ than for Ca^2+^. When Mn^2+^ binds to Fura-2, it displaces Ca^2+^, resulting in a decreased fluorescence emission signal. Fura-2 preloaded rPT cells were first treated with ouabain and monitored using time-lapse Ca^2+^ imaging. Subsequent addition of Mn^2+^ to the recording medium induced a rapid decrease in the Fura-2 emission signal (Figure 3C). Interestingly, cells that were responding to ouabain with cytosolic Ca^2+^ oscillations showed a significantly steeper decrease in Fura-2 fluorescence compared to that of non-oscillating cells (Figure 3D). These results indicate an increased amount of Ca^2+^ channels present in the PM of cells exhibiting ouabain-induced Ca^2+^ oscillations.

### Dynamic localization of PC2 is regulated by cytosolic Ca^2+^ signaling

**Figure 4:**
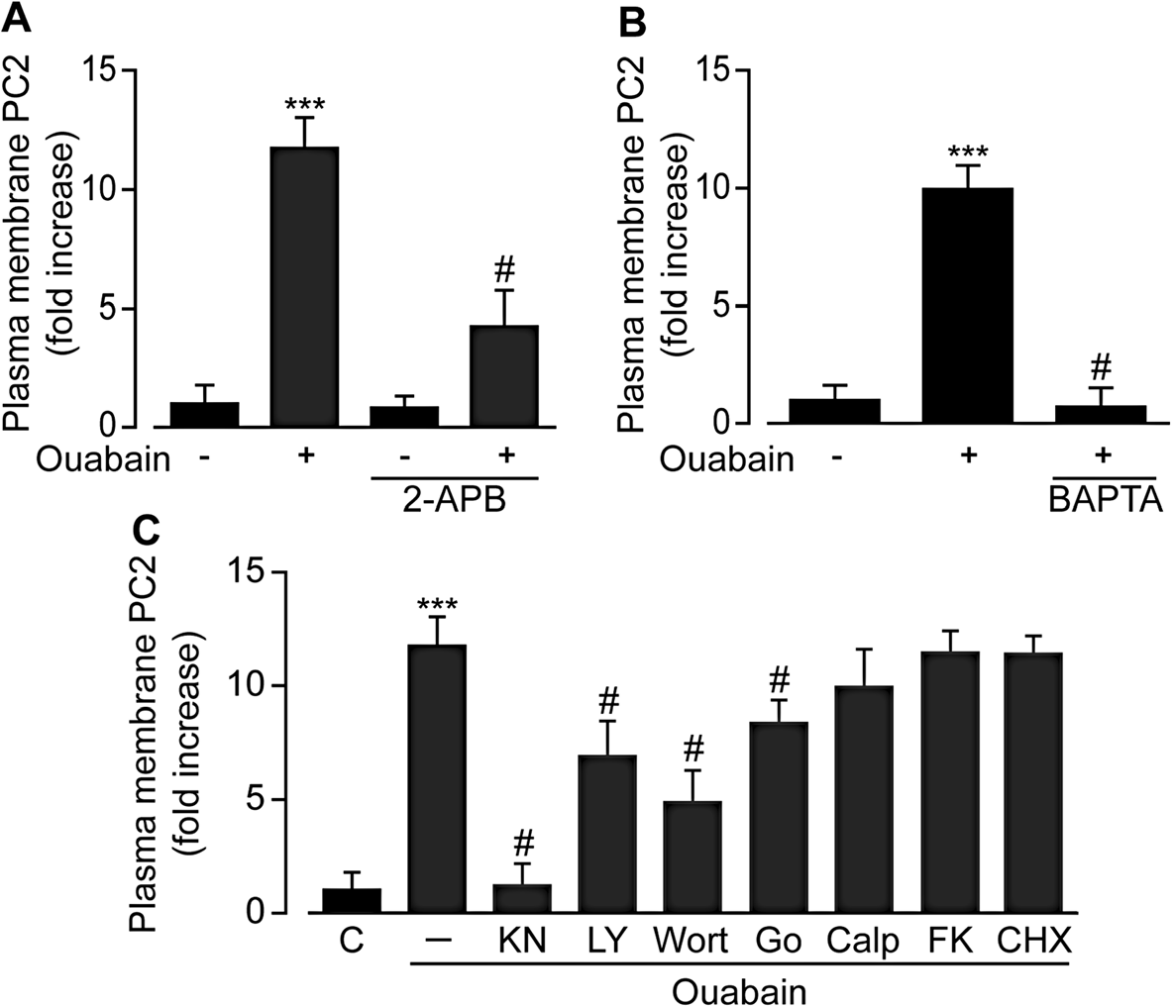
**PC2 trafficking to the PM depends on intracellular Ca**^**2+ **^**signaling and Ca**^**2+**^**-dependent kinases. (A**-**B)** Quantitative analysis of PM PC2-positive rPT cells following treatment with 100 μM ouabain with 100 μM 2-APB **(A)**, with 10 μM BAPTA **(B)**, or with 10 μM KN-93, 10 μM LY294002, 5 μM wortmanin, 20 nM FK506, 1 μM Go6983, 5 μM calphostin, or 100 μM cycloheximide **(C)**. Values are the mean ± SEM, ***P < 0.001 vs. control, and #*P* < 0.001 vs. treatment.

To further investigate the contribution of cytosolic Ca^2+^ signaling on PC2-trafficking, immunocytochemistry experiments were conducted on rPT cells stimulated with ouabain in the presence of intracellular Ca^2+^ signaling inhibitors. First, the InsP_3_R pathway was inhibited using 2-APB. In the absence of 2-APB, ouabain caused a significant 11.7 ± 1.3-fold increase of PC2 translocation to the PM (control *n* = 175 *N* = 8, ouabain *n* = 74 *N* = 4), while pre-incubation with 2-APB caused a significant 4.3 ± 1.5-fold decrease of ouabain-induced PC2 PM trafficking (ouabain *n* = 123 *N* = 7, ouabain + 2-APB *n* = 94 *N* = 4) (Figure 4A). Next, cytosolic Ca^2+^ was buffered using BAPTA. This treatment completely blocked the PC2 trafficking to the PM induced by ouabain (0.8 ± 0.7-fold, ouabain *n* = 123 *N* = 7, ouabain + BAPTA *n* = 56 *N* = 4) (Figure 4B). Together, these results suggest a pivotal role of InsP_3_R in triggering cytosolic Ca^2+^-dependent PC2 trafficking.

### Ca^2+^-dependent protein kinases are involved in dynamic PC2 localization

To investigate the involvement of Ca^2+^-sensitive kinases on PC2 trafficking, selective chemical inhibitors were used. Under control conditions, ouabain induced a significant 12.4 ± 1.3-fold translocation of PC2 to the PM (ouabain *n* = 114 *N* = 5) (Figure 4C). A common target for Ca^2+^ signaling in mammalian cells is Ca^2+^/Calmodulin-dependent kinase (CaMK). Selective inhibition of CaMK with KN93 completely blocked the ouabain-induced PC2 trafficking to the PM (1.2 ± 0.9-fold, ouabain + KN93 *n* = 104 *N* = 7) (Figure 4C). The contribution of the Phosphatidylinositol 3-kinase (PI3K)/Akt pathway was next investigated. Pre-incubation with LY294002 or wortmanin also produced a significant decrease in ouabain-induced PC2 translocation (6.9 ± 1.5-fold, ouabain + LY294002 *n* = 72 *N* = 4 and 4.8 ± 1.4-fold, ouabain + wortmanin *n* = 92 *N* = 4, respectively) (Figure 4C). Inhibition of protein kinase C (PKC) by means of broad spectrum PKC inhibitors Go6983 or calphostin resulted in decreased levels of PC2 staining in the PM that reached statistical significance only in Go6983 pre-treated cells (8.4 ± 1.0-fold, ouabain + Go6983 *n* = 117 *N* = 6 and 9.9 ± 1.7-fold, ouabain + calphostin *n* = 67 *N* = 3) (Figure 4C). Finally, inhibition of the Ca^2+^-dependent protein-phosphatase calcineurin with FK506, had no effect on the PC2 translocation (11.4 ± 1.0-fold, ouabain + FK506 *n* = 124 *N* = 7) (Figure 4C).

The observed increase in the PC2 immunosignal from the PM could be due to *di novo* synthesis of PC2 proteins. To investigate this hypothesis, cells were pre-treated with cycloheximide (CHX) to stop the translation of new proteins. Pre-treatment of cells with CHX failed to stop increased PC2 trafficking to the PM following ouabain treatment (11.4 ± 0.8-fold, ouabain + CHX *n* = 51 *N* = 3) (Figure 4C).

Together, these results suggest a translocation of cellular PC2 to the PM via the intracellular Ca^2+^/CaMK pathway, with some involvement of the PI3K/Akt and PKC pathways.

## Discussion

The notion that polycystin proteins act as cellular sensors that can modulate intracellular Ca^2+^ signaling is now well established [3]. Mounting evidence also supports the idea that mutations in the PC1 or PC2 gene perturb proper assembly, activity, and regulation of the polycystin proteins. Intriguingly, PC2 loss-of-function in modulating intracellular Ca^2+^ concentration may provide a possible explanation for the pathophysiology of ADPKD [17]. Here, we showed that the PC2 subcellular localization pattern is dynamically regulated in both human and rat kidney cells. Dependent on cytosolic Ca^2+^ increases, PC2 translocated from the cytosolic/ER compartment to the PM. When cells were challenged with ouabain, a treatment that has been shown to induce InsP_3_R-dependent Ca^2+^ oscillations in kidney cells [14,15], an increased PM PC2 localization and PM Ca^2+^ permeability were observed. The PM Ca^2+^ permeability was indirectly examined using Mn^2+^ quenching and electrophysiology recordings are required to determine absolute numbers. Pharmacological inhibition of key intracellular Ca^2+^ release components suppressed Ca^2+^-mediated PC2 trafficking, indicating a Ca^2+^-dependent translocation process. CaMK, a canonic Ca^2+^-activated kinase, was necessary for PC2 translocation, whereas the PI3K or PKC kinases contributed to a lesser extent. Mutations causing ADPKD have been reported altering the sub-cellular PC2 localization and/or function. For example, PC2 having the naturally occurring pathogenic mutant R742X resides in the PM [13], however, without having channel activity [18]. Another pathogenic missense mutation of PC2 is D511V [19], where a single amino acid in the third membrane-spanning domain is mutated, results in a loss of PC2 channel activity [3].

The subcellular localization pattern of the PC2 protein has been a long-lasting matter of controversy [20]. In various cell lines (LLCPK1, MDCK, HEK-293) and adult human kidney PC2 has been detected in ER where it function as a Ca^2+^ channel [13,21]. PC2 has also been detected in the basolateral and lateral cell membrane in adult human and rat kidney cells and collecting duct cells where it functions as cell-cell adhesion [6,21-25]. In primary cilium of human proximal tubule and mouse collecting duct cells PC2 is reported as a flow-sensitive channel [7,26,27]. These observations, and many others that report different PC2 localization [20], support the idea of a dynamic protein with its expression regulated by sub-cellular mechanisms. For example, PC2 contains an ER retention signal in its C-terminal sequence that inhibits trafficking to the cell surface [28]. PC2 deletion mutants for this ER retention signal constitutively translocate to the PM [18]. In ER Ca^2+^ stores, PC2 acts as a Ca^2+^-release channel that amplifies Ca^2+^ transients initiated by InsP_3_Rs [3]. Ca^2+^ releasing activity of PC2 is regulated by Ca^2+^ itself through a Ca^2+^-induced Ca^2+^ release mechanism [29] that requires direct association with InsP_3_R and regulates the physiological level of the intracellular Ca^2+^ concentration [30].

PC2 has previously been reported to be present in the PM [22,25]. PC2 localization to the PM is then modulated by chemical chaperones, proteasome inhibitors, protein-protein interactions and phosphorylation, and also upon massive overexpression that eventually overrides the ER retention machinery [6]. Under certain conditions, for example when PC2 is truncated at or before Glu787 its product is detected in the PM [13]. Trafficking of PC2 in cells has also been suggested to occur through its physical interaction with PC1 via their C-termini, forming a heteromeric, non-selective cationic channel complex [31]. Efficient assembly of PC1 and PC2 appears to be essential for proper trafficking and channel activity [32]. These previous results and the findings presented here support the idea of a dynamically regulated subcellular localization of PC2.

Our results indicate that CaMK is an important regulator of PC2 trafficking to the PM. Indeed, phosphorylation has previously been demonstrated in PC2 trafficking [13]. Two evolutionarily conserved phosphorylation sites in the PC2 protein sequence were suggested to control its subcellular localization: serine residue 76 (Ser76)/Ser80 and Ser812, which are phosphorylated by glycogen synthase kinase 3 (GSK3) [33] and casein kinase 2 (CK-2) [34], respectively. We speculate that the PM PC2 portion might be phosphorylated differently than ER PC2. It has been shown that PKC-dependent phosphorylation at Ser801 is essential for a normal function of PC2 as an ER Ca^2+^ release channel [35]. Additionally, Never in mitosis A-related kinase 8 (Nek8), a serine/threonine kinase that is mutated in some cases of juvenile polycystic kidney disease, induced abnormal PC2 phosphorylation and trafficking to primary cilia in the kidney [36]. The Ca^2+^ dependent trafficking of PC2 reported herein might also play a role in the PC2 expression profile in cilia. Surprisingly little is known about the upstream physiological stimuli activating all these kinases to critically regulate PC2 trafficking. Whether the Ca^2+^ signaling pathway presented here is the missing upstream link in controlling CaMK, GSK-3, CK-2 or Nek8 in PC2 trafficking remains to be further examined.

Polycystin proteins are expressed in primary cilia of cultured renal epithelial cells, where they might function in transducing sensory information, such as shear stress during fluid flow [7], leading to Ca^2+^ influx through mechanically sensitive channels that reside in the ciliary membrane [37]. This Ca^2+^ signal is then amplified by Ca^2+^ release from internal ER/SR stores and spreads to neighboring cells through gap junctions. PC2 has been suggested to be a mechano-sensitive channel since mechano-transduction is abolished in the presence of a specific PC2 blocking antibody [7] and in epithelial cells isolated from PC1-deficient mice [7]. Intracellular Ca^2+^ release and PC2 trafficking to the PM may form a positive loop for Ca^2+^ influx and intracellular Ca^2+^ overload, a condition that has been reported previously in ADPKD progression [1,5]. However, the exact contribution of PC2 dynamic localization to the PM in ADPKD pathogenesis remains to be elucidated.

Our results show that endogenous agonists that increase cytosolic Ca^2+^ levels, such as ouabain, ATP, or bradykinin, induced PC2 trafficking to the PM. It was previously reported that ouabain triggers InsP_3_R-dependent intracellular Ca^2+^ oscillation in cultured rPT cells [14,15]. According to the results presented here, cells that exhibited ouabain-induced Ca^2+^ oscillations had increased PM Ca^2+^ permeability. Other reports have shown that cilia mechano-activation leads to ATP secretion, which acts as an auto/paracrine signal through purinergic receptor activation and intracellular Ca^2+^ signals [38], suggesting that both agonists may contribute to PC2 translocation to the PM under physiopathological conditions.

## Conclusion

We conclude that PC2 subcellular localization is dynamically regulated through an intracellular Ca^2+^-dependent pathway, which in turn could be related to cystogenesis and ADPKD pathogenesis.

## Competing interests

The authors declare that they have no competing interests.

## Authors’ contributions

AM conceived the study and participated in its design, carried out the immunocytochemistry studies, cultured cells and drafted the manuscript. CI performed the statistical analysis and drafted the manuscript. SM carried out the manganese quenching experiments. AA conceived the study and participated in its design. PW participated in the design of the study and coordinated the collection of human tissue. PU designed the study and wrote the manuscript. All authors read and approved the final manuscript.

## Pre-publication history

The pre-publication history for this paper can be accessed here:

http://www.biomedcentral.com/1471-2369/14/34/prepub

## Supplementary Material

Additional file 1: Figure S1 PC2 expression pattern in proximal tubule cells using two different antibodies. (A-D) Immunocytochemistry of PC2 in rat proximal tubule cells treated with control (A,C) or 100 μM ouabain (B,D) using anti-PC2 polyclonal antibodies against amino acids 103 to 203 (YCB9) or 44 to 62 on the N-terminus. Scale bars, 20 μm. Click here for file

Additional file 2: Figure S2 Immunocytochemistry negative control without primary PC2 antibody. (A-B) Immunocytochemistry staining in rat proximal tubule cells without (A) and with (B) anti-PC2 antibody present. Scale bars, 20 μm. Click here for file
