# Supplementary figures and images for: Intracellular calcium release modulates polycystin-2 trafficking
Source: BMC Nephrol. 2013 Feb 11;14:34. doi: 10.1186/1471-2369-14-34 (PMC3577431; doi:10.1186/1471-2369-14-34)

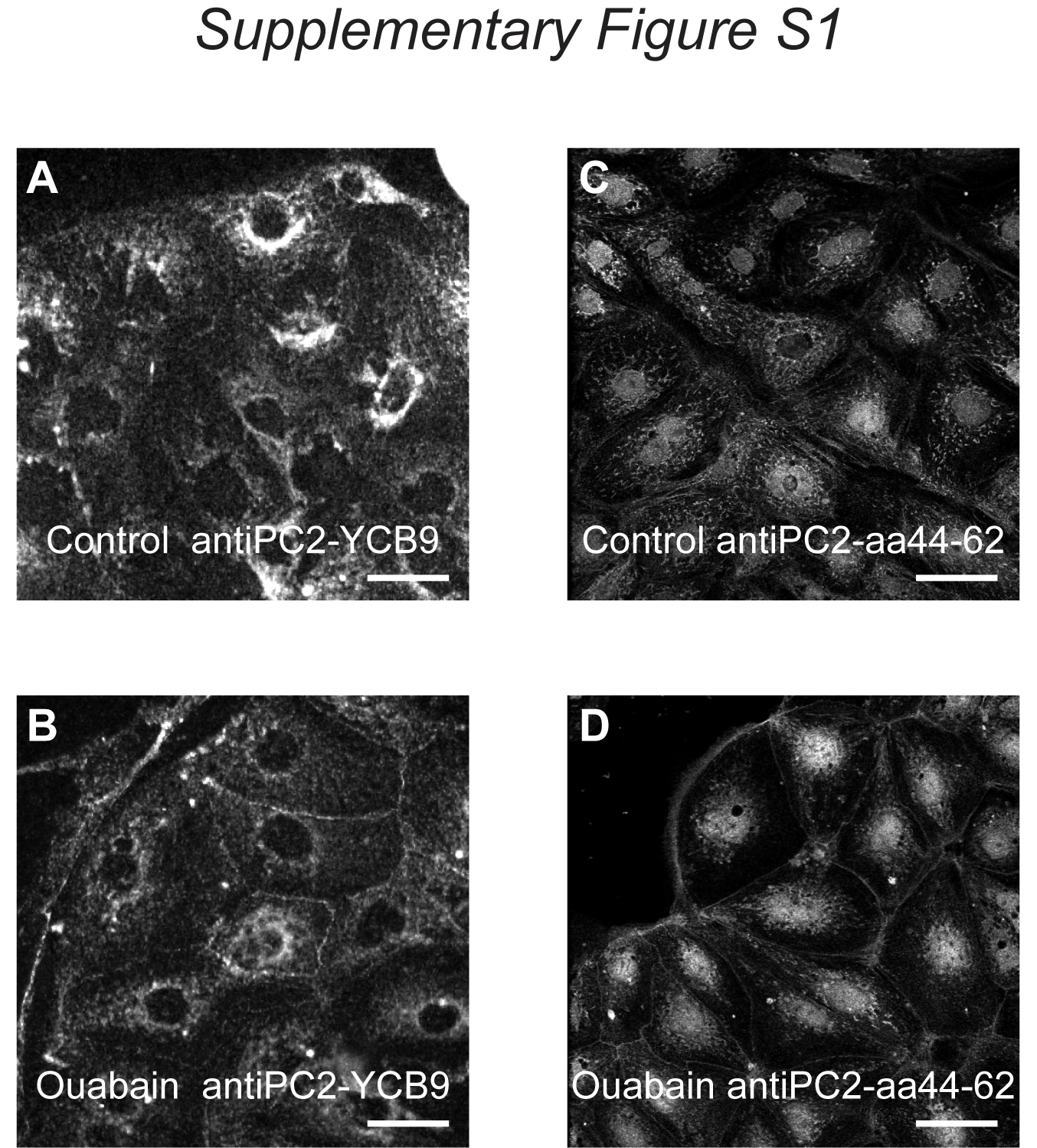

Supplement: Additional file 1: Figure S1 — PC2 expression pattern in proximal tubule cells using two different antibodies. (A-D) Immunocytochemistry of PC2 in rat proximal tubule cells treated with control (A,C) or 100 μM ouabain (B,D) using anti-PC2 polyclonal antibodies against amino acids 103 to 203 (YCB9) or 44 to 62 on the N-terminus. Scale bars, 20 μm. [file 1471-2369-14-34-S1.tiff]

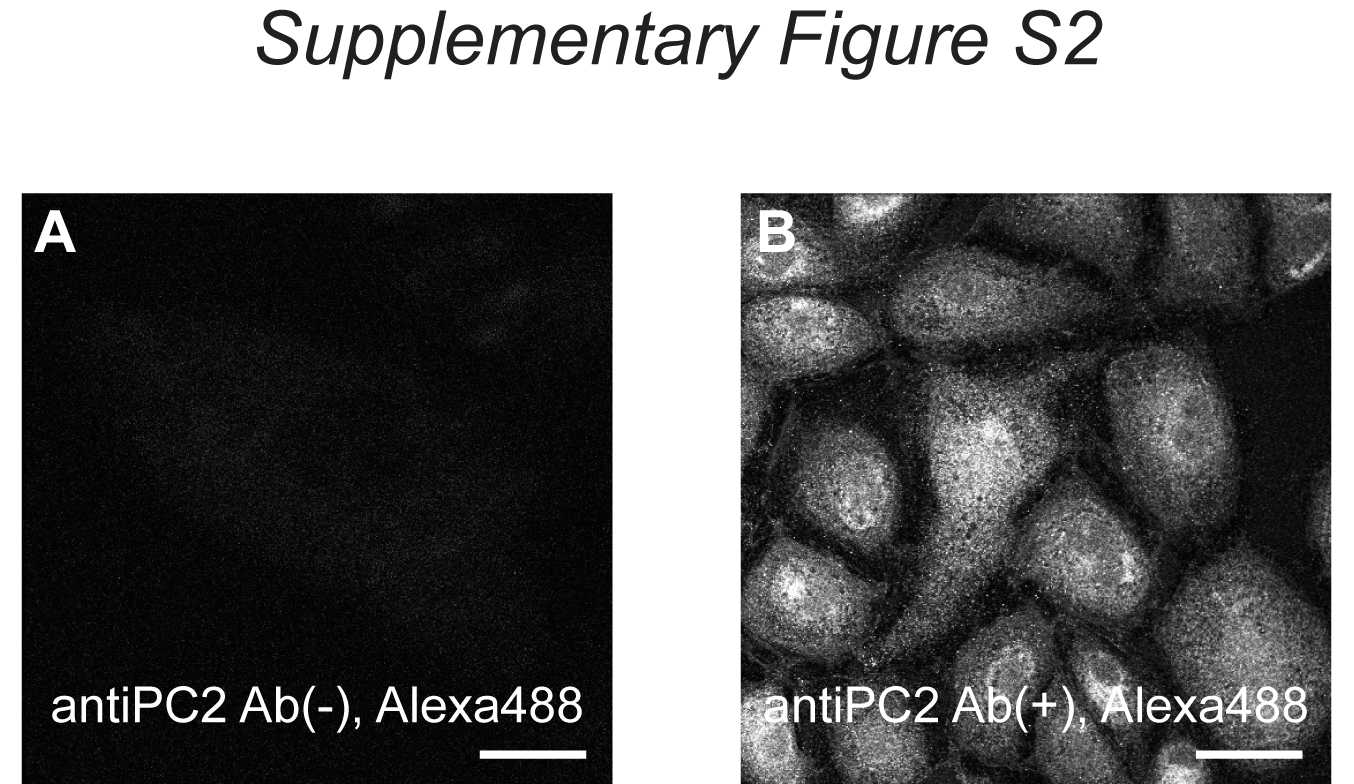

Supplement: Additional file 2: Figure S2 — Immunocytochemistry negative control without primary PC2 antibody. (A-B) Immunocytochemistry staining in rat proximal tubule cells without (A) and with (B) anti-PC2 antibody present. Scale bars, 20 μm. [file 1471-2369-14-34-S2.tiff]
